# Oxidative and Inflammatory Imbalance in Placenta and Kidney of sFlt1-Induced Early-Onset Preeclampsia Rat Model

**DOI:** 10.3390/antiox11081608

**Published:** 2022-08-19

**Authors:** Álvaro Santana-Garrido, Claudia Reyes-Goya, Pablo Espinosa-Martín, Luis Sobrevia, Luis M. Beltrán, Carmen M. Vázquez, Alfonso Mate

**Affiliations:** 1 Departamento de Fisiología, Facultad de Farmacia, Universidad de Sevilla, 41012 Sevilla, Spain; 2 Epidemiología Clínica y Riesgo Cardiovascular, Instituto de Biomedicina de Sevilla (IBIS), Hospital Universitario Virgen del Rocío/Consejo Superior de Investigaciones Científicas/Universidad de Sevilla, 41013 Sevilla, Spain; 3 Cellular and Molecular Physiology Laboratory (CMPL), Department of Obstetrics, Division of Obstetrics and Gynaecology, Pontificia Universidad Católica de Chile, Santiago 8330024, Chile; 4 Medical School (Faculty of Medicine), São Paulo State University (UNESP), São Paulo 01049-010, Brazil; 5 University of Queensland Centre for Clinical Research (UQCCR), Faculty of Medicine and Biomedical Sciences, University of Queensland, Herston, QLD 4029, Australia; 6 Department of Pathology and Medical Biology, University of Groningen, University Medical Center Groningen (UMCG), 9713 GZ Groningen, The Netherlands; 7 Tecnológico de Monterrey, Eutra, The Institute for Obesity Research (IOR), School of Medicine and Health Sciences, Monterrey 64710, Nuevo León, Mexico; 8 Departamento de Medicina, Facultad de Medicina, Universidad de Sevilla, 41009 Sevilla, Spain

**Keywords:** inflammation, kidney, NADPH oxidase, nitric oxide, oxidative stress, placenta, preeclampsia, sFlt1

## Abstract

Preeclampsia (PE) is a pregnancy-specific disorder characterized by the new onset of hypertension plus proteinuria and/or end-organ dysfunction. Here, we investigate the role of the nicotinamide adenine dinucleotide phosphate (NADPH) oxidase system as a major component of reactive oxygen species generation, in a rodent model of early-onset preeclampsia induced by excess sFlt1 (soluble fms-like tyrosine kinase 1). Placenta and kidney samples were obtained from normal pregnant and PE rats to measure the sFlt1/PlGF (placental growth factor) ratio in addition to oxidative stress-related parameters, including the activities and expressions of NADPH oxidase isoforms (NOX1, NOX2, and NOX4), components of nitric oxide (NO) metabolism, and antioxidant enzymes. Peroxisome proliferator-activated receptors (PPARα, PPARγ) and cytokines IL1β, IL3, IL6, IL10, and IL18 were also measured to evaluate the inflammation status in our experimental setting. Excessive O_2_^●−^ production was found in rats that were treated with sFlt1; interestingly, this alteration appears to be mediated mainly by NOX2 in the placenta and by NOX4 in the kidney. Altered NO metabolism and antioxidant defense systems, together with mitochondrial dysfunction, were observed in this model of PE. Preeclamptic animals also exhibited overexpression of proinflammatory biomarkers as well as increased collagen deposition. Our results highlight the role of NADPH oxidase in mediating oxidative stress and possibly inflammatory processes in the placenta and kidney of an sFlt1-based model of early-onset preeclampsia.

## 1. Introduction

Preeclampsia (PE) is a gestational hypertensive syndrome that affects around 3–10% of pregnancies worldwide [1]. According to current guidelines by the International Society for the Study of Hypertension in Pregnancy (ISSHP), this syndrome is described by new-onset hypertension that can be accompanied by proteinuria and/or other maternal end-organ damage at ≥20 weeks of gestation [2]. Preeclampsia causes almost 15% of maternal deaths every year [3], postulating itself as one of the main causes of maternal mortality, neonatal and fetal mortality, and preterm birth. The risk of PE can be reduced following healthy lifestyle habits, such as adequate maternal nutrition and moderate physical activity, or by the absence of medical complications including diabetes mellitus or viral infections [4,5]. PE is a multisystem disorder that can affect the placenta, kidney, heart or liver, among other organs [6,7], thus increasing the risk of developing cardiovascular, renal, and neural diseases in both mother and child [8,9,10]. PE is generally subdivided into early-onset and late-onset types based on gestational age at diagnosis [11] (<34 or ≥34 weeks, respectively); in any case, both forms are associated with an increase in the antiangiogenic marker sFlt1 (soluble fms-like tyrosine kinase 1) in maternal serum [2]. The intrinsic cellular and molecular mechanisms that trigger this disorder and contribute to disease progression remain unknown; as a consequence, early diagnosis and specific treatments have not yet been fully implemented.

The presence of placental dysfunction and the alleviation of symptoms after placental delivery point to this organ as the key core in the development of PE. Inaccurate trophoblast invasion with subsequent defective remodeling of the spiral artery [12], followed by an incomplete neovascular process resulting in placental vascular dysfunction, are hallmarks of the “classic” form of PE. In this sense, oxidative stress has been postulated as one of the factors responsible for this placental dysfunction [13]. Moreover, the interplay between oxidative stress and inflammatory processes results in an impairment of vascular homeostasis that aggravates the development of endothelial dysfunction. The high production of reactive oxygen species (ROS) [14] affects the activity and expression of endothelial nitric oxide synthase (eNOS) enzyme [15], with a reduction in nitric oxide (NO) bioavailability in PE. 

Previous reports support the notion that ROS production (mostly superoxide anion, O_2_^●−^) in the placenta is primarily accomplished by the enzyme nicotinamide adenine dinucleotide phosphate (NADPH) oxidase, or NOX [16,17]. The catalytic subunit of this enzyme includes at least seven isoforms (NOX1-5 and Duox1-2) [18]. ROS generation via NADPH oxidase may be responsible for NO depletion, leading to eNOS uncoupling and excess O_2_^●−^ production that worsen the situation of oxidative imbalance. Although NADPH oxidase has been previously suggested as a relevant source of ROS in the pathogenesis of PE [17], few studies have explored the regulation of specific NOX isoforms in the placenta and other target organs in this context. 

The purpose of this study was to investigate the role of the NADPH oxidase system as a key element responsible for ROS generation in a rodent model of early-onset PE induced by sustained elevation of sFlt1 circulating levels. PE-like syndrome was created by using osmotic mini-pumps loaded with sFlt1 that were implanted intraperitoneally from gestation day (GD) 7 to GD19. In order to confirm successful induction of the disorder, blood pressure, proteinuria, and sFlt1/PlGF (placental growth factor) ratio were routinely measured throughout the study. The degree of oxidative stress was estimated by measuring O_2_^●−^ levels, nitrosylation of proteins, the activity and expressions of NOX isoforms, and the participation of additional sources of O_2_^●−^ other than NADPH oxidase, in the placenta and kidney of normal pregnant and preeclamptic rats. The activity and expressions of antioxidant enzymes (namely, superoxide dismutase, SOD; glutathione peroxidase, GSH-Px; and glutathione reductase, GSH-Red), as well as an evaluation of NO metabolism (including an estimation of NO levels, the expressions of arginases (Arg-1 and Arg-2), and that of eNOS) were also assayed in the same tissues in both experimental groups. In addition, peroxisome proliferator-activated receptor (PPAR) isoforms (PPARα and PPARγ) and interleukins (IL1β, IL3, IL6, IL10, and IL18) were quantified as markers of the inflammation process that might be associated with NADPH oxidase-dependent ROS overproduction. 

## 2. Materials and Methods

### 2.1. Study Design

All animal protocols were conducted in accordance with the European Union (EU) Directive 2010/63/EU, as well as the National (RD 53/2013) Guidelines for the Care and Use of Laboratory Animals, and were approved by the competent Institutional Animal Care and Use Committee (approval reference #31/05/2021/090, issued by Junta de Andalucía, Dirección General de la Producción Agrícola y Ganadera). Female Wistar rats aged 10–12 weeks were obtained from the Center for Animal Production and Experimentation at the University of Seville (Spain). The animals were randomly assigned into two groups: (1) control animals undergoing a normal pregnancy (NP group, *n* = 8) and (2) animals with early-onset preeclampsia induced as described below (PE group). The presence of vaginal plugs defined the first gestational day (GD0). All animals were housed in a regulated environment under standard conditions (23 ± 1 °C, 12 h/12 h light/dark cycles).

### 2.2. Preeclampsia Induction

Early-onset preeclampsia was induced using Alzet^®^ osmotic mini-pumps (DURECT Corporation, Cupertino, CA, USA, mods. 2002, 1003D) loaded with sFlt1 (recombinant mouse VEGFR type 1/Flt1 Fc chimera; R&D Systems, Minneapolis, MN, USA, cat # 7756-FL). Mini-pumps were implanted intraperitoneally in animals that were anesthetized by controlled isoflurane inhalation vapour (3%) on gestation day (GD) 7, and maintained until GD19. During this period, sFlt1 was infused at a rate of 3.7 µg/kg/day. Samples were processed at GD19 (PE-19 group, *n* = 8), except for specific experiments performed in an additional group of animals (*n* = 6) that were sacrificed at GD11 (PE-11 group, i.e., sFlt1 infused from GD7 to GD11) to study potential signs of early renal damage.

### 2.3. Animal Characteristics

Following previous protocols established in our laboratory, systolic and diastolic blood pressure values were measured on GD5 (baseline), GD11, and GD19 using an NIPREM 645 pressure recorder (CIBERTEC S.A., Madrid, Spain). Proteinuria was measured using 24-h urine samples that were collected in individual metabolic cages between GD10/GD11 or between GD18/GD19. Protein concentration was evaluated using the Bradford method [19].

### 2.4. Sample Harvesting and Tissue Homogenization

Rats were deeply anesthetized by 5% isoflurane inhalation. Blood was rapidly collected by cardiac puncture. Fetuses and placentas were counted in both uterine horns and the latter were collected without umbilical cords. The kidneys were decapsulated, and then the renal cortex was dissected apart. Both placentas and renal cortexes were snap-frozen in liquid nitrogen and stored at −80 °C until use. Tissue homogenates were prepared in 50-millimolar phosphate buffer saline (PBS, pH 7.4) that contained protease inhibitors (Sigma Aldrich-Roche, Madrid, Spain) following routine protocols in our laboratory [20], and the protein concentration in these homogenates was determined using the Bradford method [19].

### 2.5. Determination of sFlt1, PlGF and NO Levels

Mouse Quantikine ELISA kits purchased from R&D Systems were used to measure the concentrations of sFlt1 (cat. # MVR100) and PlGF (cat. # MP200) in placental homogenates obtained as described above and following the manufacturer’s protocols. In turn, placental and kidney homogenates were used to estimate nitric oxide concentration via a nitrite assay kit from Sigma-Aldrich (Merck Life Science S.L.U., Madrid, Spain, cat. # MAK367).

### 2.6. Histological Staining and Superoxide Anion Levels

5-micrometer paraffin sections were obtained as previously reported [21]. These sections were used for collagen fiber deposition analysis and for quantification of superoxide anion production *in situ*. Collagen fibers were evaluated by picro Sirius Red staining (SRS; (Sigma-Aldrich, cat. # 365548)), as described elsewhere [22]. Superoxide anion production was measured by using the fluorescent dye dihydroethidium (DHE; MedChemExpress, cat. # HY-D0079) [21]. This molecule specifically reacts with intracellular superoxide anion and turns into ethidium (a red fluorescent compound) in nuclei. In order to confirm the specificity of O_2_^●−^ measurement, placenta and kidney slides were preincubated with 100 U/mL polyethylene glycol-conjugated superoxide dismutase (PEG-SOD; Sigma Aldrich, cat. # S9549) following regular protocols [21]. Since PEG-SOD can act as a superoxide scavenger, ablation of the fluorescent signal after incubation indicates DHE specificity, while the remaining red signal indicates background [23]. Image J-NIH freeware (v. 2.0.0) was used to quantify the intensity of the staining.

### 2.7. NADPH Oxidase Activity

Nicotinamide adenine dinucleotide phosphate (NADPH) oxidase activity and superoxide anion (O_2_^●−^) sources were measured in placenta and renal cortex homogenates by lucigenin-enhanced chemiluminescence [21]. Samples were preincubated for 5 min at 37 °C with the following inhibitors of potential O_2_^●−^sources (0.1 mmol/L; Sigma-Aldrich): DPI (inhibitor of flavoproteins); oxypurinol (inhibitor of xanthine oxidase); and rotenone (inhibitor of the mitochondrial electron transport chain). Different inhibitors of NADPH oxidase (Sigma-Aldrich) were also used as follows to explore the relative contributions of each NOX isoform in O_2_^●−^ production: specific NOX1 inhibitor (0.5 µmol/L, ML171, cat. # 175226); NOX1/4 inhibitor (0.1 µmol/L, GKT136901, cat. # 492000); and a pan-NADPH oxidase inhibitor (10 µmol/L, VAS2870, cat. # 5340320001). All measurements were normalized to the protein content in the samples, and results were expressed in relation to the NP group.

### 2.8. Antioxidant Enzyme Activities

Superoxide dismutase (SOD), glutathione peroxidase (GSH-Px) and glutathione reductase (GSH-Red) activities were assayed following the manufacturer’s protocols for each enzyme (Cayman Chemical Company, Ann Arbor, MI, cat. # 706002-96, 703102-96, and 703202-96, respectively).

### 2.9. Real-Time PCR

Total RNA was isolated from placental and renal tissues using the Trizol^®^ method (Thermo Fisher Scientific, Madrid, Spain). Then, reverse transcription reactions were performed as described elsewhere [24]. Real-time PCR analyses were conducted using the specific primers listed in Table 1. Gene products were amplified in a CFX96 real-time PCR system (Bio-Rad, Madrid, Spain). Glyceraldehyde-3-phosphate dehydrogenase (GAPDH) was used as a housekeeping gene to quantify the relative changes in mRNA expressions following the standard 2^−ΔΔCt^ method.

### 2.10. Western Blotting

Placental and kidney homogenates (equivalent to 60–80 µg of proteins) were subjected to SDS-PAGE and immune-blotted with specific antibodies (Table 2), as previously described [25]. Quantification analyses were performed with an Amersham Imager 600 blot and gel imaging instrument, using β-actin protein as a loading control.

### 2.11. Statistical Analyses

Results were analyzed using GraphPad Prism version 5.1 (GraphPad Software Inc. San Diego, CA, USA, 2007) and expressed as means ± SEM. The unpaired two-tailed Student’s *t*-test was run, and differences were considered statistically different at *p* < 0.05. When comparing more than two groups, one-way ANOVA followed by Tukey’s multiple comparison test were performed.

## 3. Results

### 3.1. General Characteristics of the sFlt1-Dependent Animal Model of Preeclampsia

**Figure 1:**
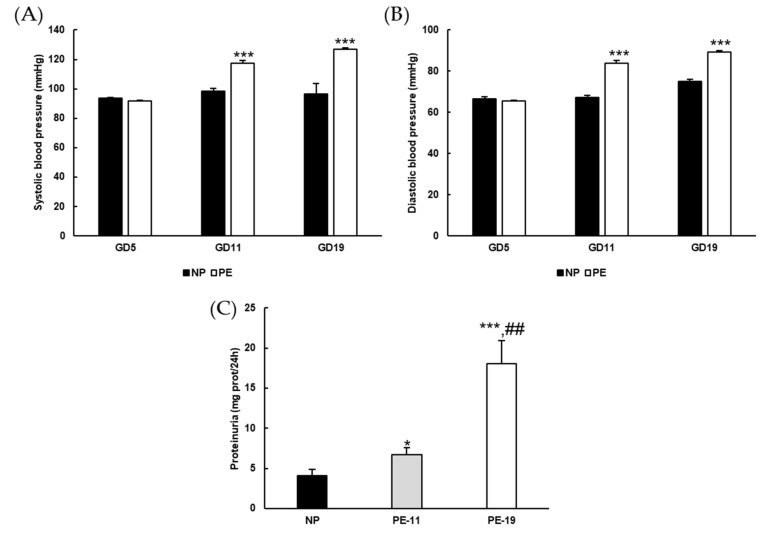
**General parameters.** (**A**) Systolic and (**B**) diastolic blood pressure values recorded at gestation days (GD) 5, GD11 and GD19, and (**C**) protein concentration in 24-h urine samples in the different experimental groups. Values are expressed as mean ± SEM of at least six animals per group: * *p* < 0.05, *** *p* < 0.001 vs. NP; ^##^ *p* < 0.01 vs. PE-11. NP: normal pregnant rats; PE: preeclamptic rats; PE-11: preeclamptic rats at GD11; PE-19: preeclamptic rats at GD19.

Blood pressure (BP) was significantly higher in the PE group than in the NP group from GD11 to the end of the treatment, with respective increases of ~30 and ~14 mmHg for systolic and diastolic BP (Figure 1A,B). Urinary protein excretion revealed the presence of considerable proteinuria in preeclamptic rats (Figure 1C), despite no changes in dietary behavior (data not shown). The average placental weight was reduced by ~0.24 g in the PE group, an alteration that was not found in the kidneys. The number of embryos was similar but with low weight in the PE group (Table 3).

### 3.2. Molecular Characterization of the sFlt1-Based Preeclampsia Animal Model

**Figure 2:**
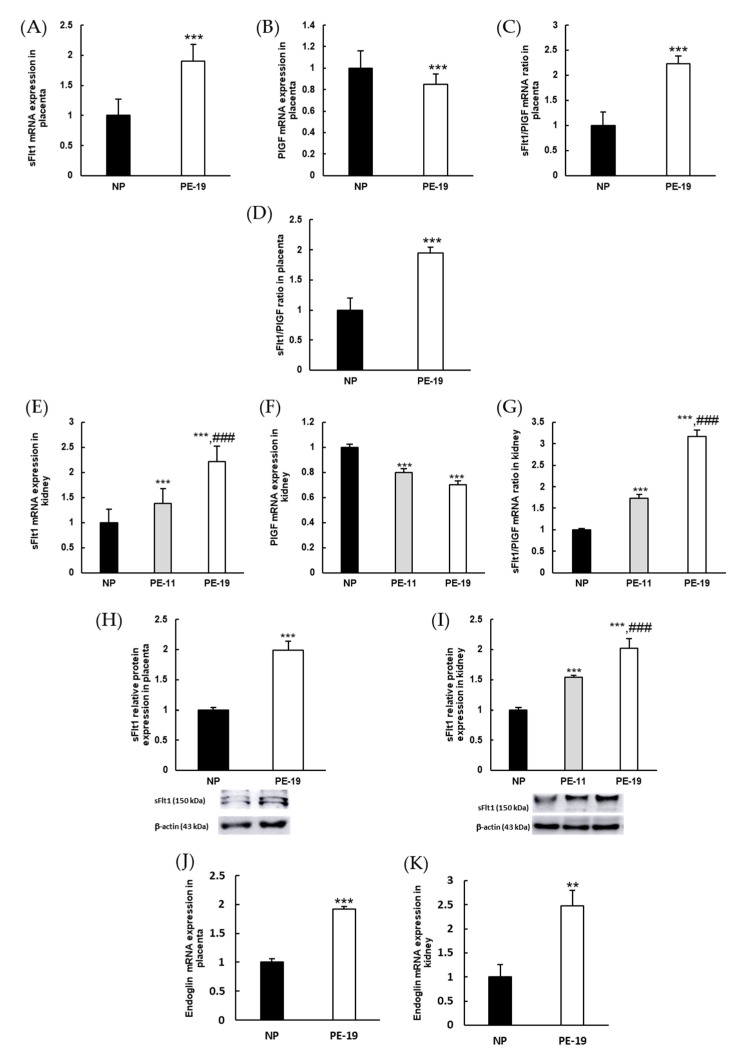
**Molecular characterization of preeclampsia.** Gene expressions of (**A**) sFlt1 and (**B**) PlGF as well as (**C**) sFlt1/PlGF mRNA ratios in the placentas of NP and PE-19 rats. (**D**) The sFlt1/PlGF ratio was measured by immunoassay in placental homogenates. (**E**–**G**) Similar experiments as in (**A**–**C**) were performed in the kidney, including an additional (PE-11) preeclamptic group. (**H**,**I**) sFlt1 protein expressions and (**J**,**K**) endoglin gene expressions in placenta and kidney, respectively. The quantitative fold changes in gene expressions were determined relative to GAPDH in each corresponding group. Values are expressed as mean ± SEM of at least six animals per group: ** *p* < 0.01, *** *p* < 0.001 vs. NP; ^###^ *p* < 0.001 vs. PE-11. NP: normal pregnant rats; PE-11: preeclamptic rats at GD11; PE-19: preeclamptic rats at GD19.

Besides the presence of hypertension and proteinuria, the induction of a preeclampsia-like syndrome was confirmed by measuring the levels of sFlt1 and PlGF in the harvested tissues. In placentas, the sFlt1/PlGF ratio increased by two-fold in PE-19 animals, both when estimated from the relative mRNA expression (Figure 2C) and by immunoassay (Figure 2D). Similar results were found in renal cortex homogenates, where the sFlt1/PlGF mRNA ratio was about three times higher in PE-19 than in NP rats (Figure 2G). Interestingly, this parameter was also significantly augmented in the PE-11 group (1.73-fold change), confirming the imbalance of angiogenic factors in the kidney from GD11 (i.e., after four days of sFlt1 infusion). Furthermore, sFlt1 protein expressions doubled in placental and renal homogenates obtained from PE-19 rats; these were already evident in the kidney at GD11 (Figure 2H,I). Additionally, preeclampsia induced up-regulation of endoglin gene expression in both tissues (1.92-fold and 2.48-fold changes in the placenta and kidney, respectively; Figure 2J,K).

### 3.3. Collagen Deposition in the Placenta and Kidney

**Figure 3:**
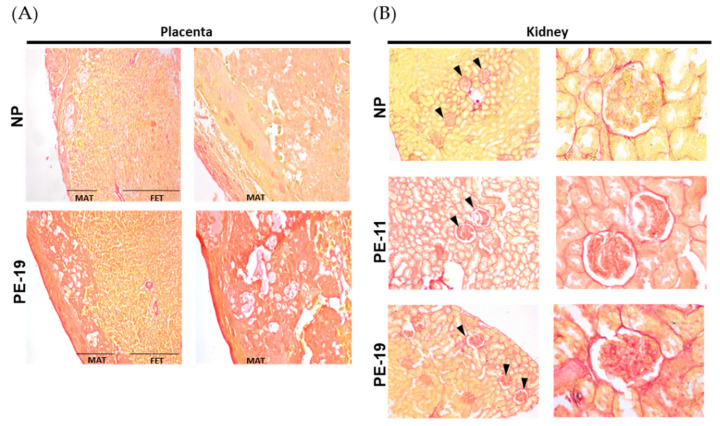
**Collagen deposition in placenta and kidney**. Representative pictures of (**A**) placenta (left) and (**B**) kidney (right) slices stained with Sirius Red dye. Images are organized into two columns per animal group with magnifications as follows: 4× (left column) and 10× (right column) for placenta, and 10× (left column) and 40× (right column) for kidney samples. In the kidney, glomeruli are indicated by black arrowheads. NP: normal pregnant rats; PE-11: preeclamptic rats at GD11; PE-19: preeclamptic rats at GD19; MAT: maternal portion of the placenta; FET: fetal portion of the placenta.

Sirius Red-stained placentas from PE animals revealed a higher reddish coloration in comparison with the NP group, both in the maternal (MAT) and fetal (FET) portions (Figure 3A). Similarly, the intensity of red staining was higher in renal corpuscles and tubules from PE-11 and PE-19 rats compared to NPs (Figure 3B).

### 3.4. Preeclampsia-Induced O_2_^●−^ Overproduction and Nitrosylation of Proteins in Placental and Renal Tissues

**Figure 4:**
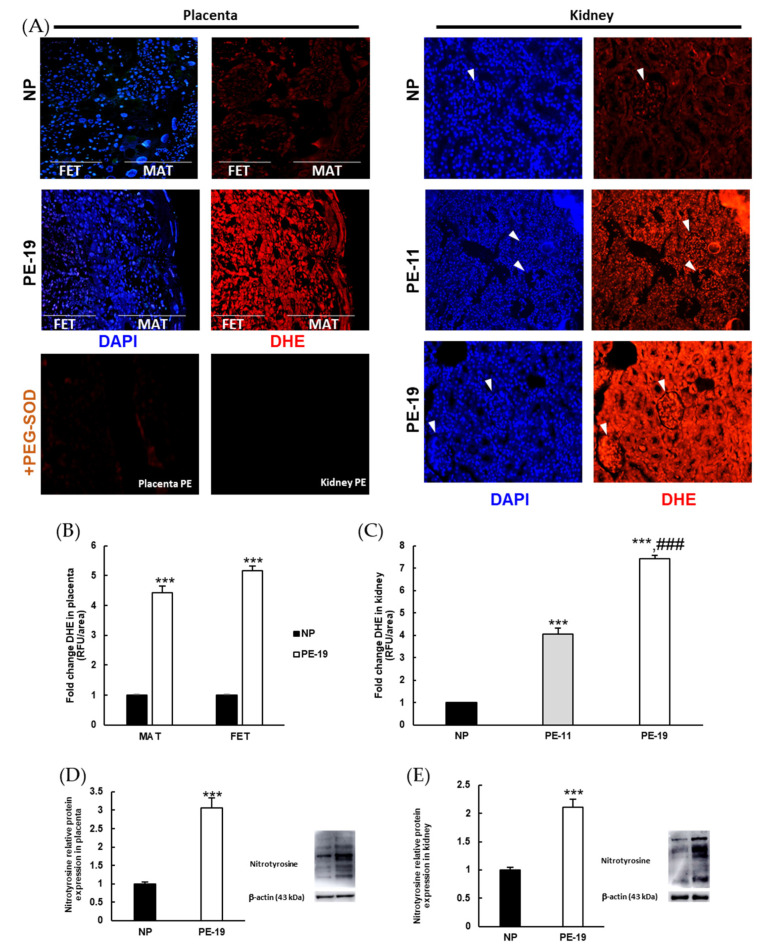
**Superoxide levels and protein nitrosylation in the placenta and kidney.** (**A**) Representative images of dihydroethidium (DHE) labelling (red color) for superoxide anion (O_2_^●−^) in the placentas (left panels) and kidneys (right panels) from normal pregnancies and preeclamptic rats. Nuclei can be distinguished with 4′,6-diamidino-2-phenylindole (DAPI, blue color) staining. In the kidney, glomeruli are indicated by white arrowheads. The effect of preincubation with polyethylene glycol-conjugated superoxide dismutase (PEG-SOD) in placenta and kidney slices is shown at the bottom left. Magnification: 4×. (**B**,**C**) Quantification of DHE staining intensity (relative to NP group) by Image J software in the placenta and kidney, respectively. (**D**,**E**) Protein nitrosylation estimated by Western blotting in placenta and renal cortex homogenates, respectively. Values are expressed as mean ± SEM of at least six animals per group: *** *p* < 0.001 vs. NP; ^###^ *p* < 0.001 vs. PE-11. NP: normal pregnant rats; PE-11: preeclamptic rats at GD11; PE-19: preeclamptic rats at GD19. MAT: maternal portion of the placenta; FET: fetal portion of the placenta; RFU: relative fluorescence units.

The DHE fluorescence assay detects nuclear O_2_^●−^ through a chemical reaction between superoxide and DHE, whereby the latter is oxidized to ethidium [23]. An intensified DHE signal (red color) was found in the placenta and kidney of PE groups when compared with the NP group (Figure 4A). The abolition of O_2_^●−^ production in the presence of PEG-SOD confirmed the specificity of DHE staining in both organs (Figure 4A, bottom). Fluorescence signals were significantly increased in the PE-19 group in both the maternal and the fetal portions of the placenta (4.42-fold and 5.17-fold, respectively; Figure 4B). In the kidney, superoxide-dependent DHE staining was similar in renal corpuscles and tubules, showing intensified signals in the PE-11 and PE-19 groups relative to NP animals (Figure 4C). The degree of protein nitrosylation, measured as another marker of oxidative stress, also revealed significant alterations both in the placenta (3.06-fold increase) and in the kidney (2.11-fold increase) of sFlt1-treated rats (Figure 4D,E).

### 3.5. NOX Activation and Alterations in NO Metabolism Are Involved in the Oxidative Imbalance Present in the sFlt1-Dependent Model of Preeclampsia

**Figure 5:**
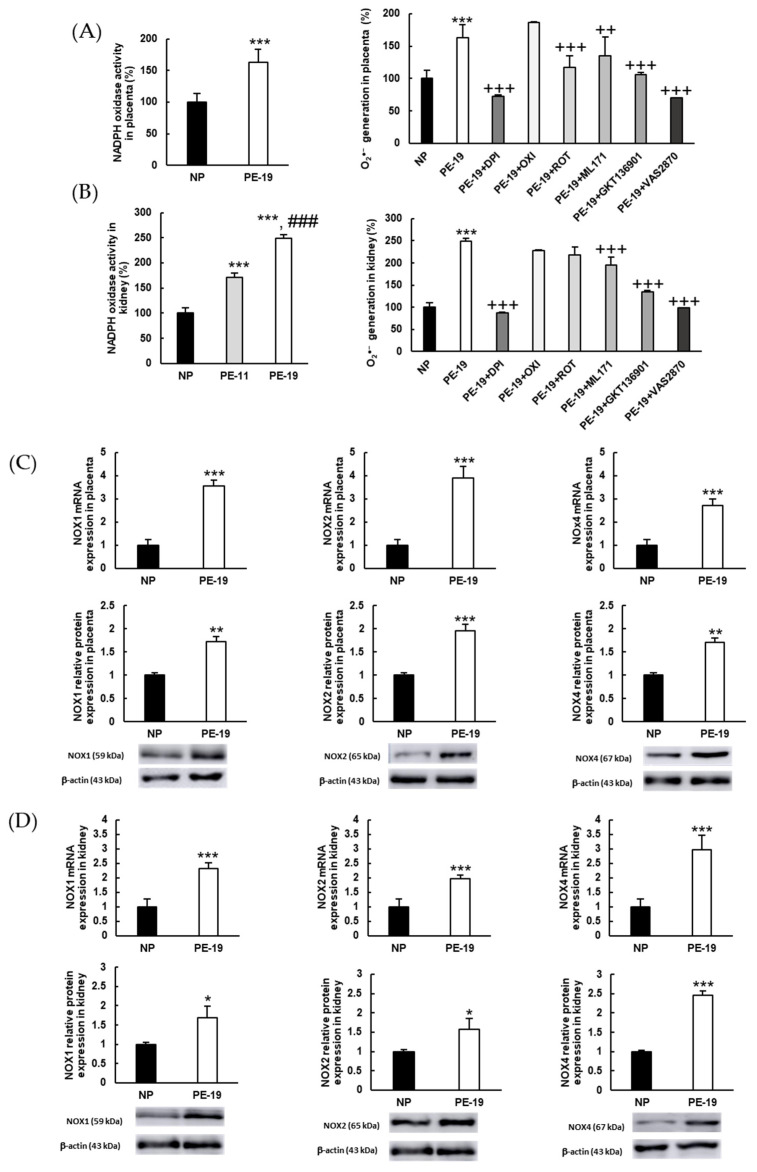
**Superoxide sources and activities/expressions of NADPH oxidase (NOX) isoforms in the placenta and kidney.** (**A**,**B**) NADPH oxidase activities (left charts) and characterization of the primary sources of O_2_^●−^ (right charts) in placenta and renal cortex homogenates, respectively. Preincubation with different inhibitors was performed as detailed in Section 2.7: DPI: diphenyleneiodonium chloride (inhibitor of flavoproteins); OXI: oxypurinol (xanthine oxidase inhibitor); ROT: rotenone (inhibitor of the mitochondrial electron transport chain); ML171: NOX1 inhibitor; GKT136901: dual NOX1/NOX4 inhibitor; VAS2870: NOX enzyme pan-inhibitor. (**C**) Gene (upper charts) and protein (lower charts) expressions of NOX1, NOX2, and NOX4 in placental homogenates. (**D**) Similar experiments as in (**C**) performed in renal cortex homogenates. The quantitative fold changes in gene expressions were determined relative to the corresponding values for the glyceraldehyde-3-phosphate dehydrogenase (GAPDH) housekeeping gene. Values are expressed as mean ± SEM of at least six animals per group: * *p* < 0.05, ** *p* < 0.01, *** *p* < 0.001 vs. NP; ^###^ *p* < 0.001 vs. PE-11; ^++^ *p* < 0.01, ^+++^ *p* < 0.001 vs. PE-19. NP: normal pregnant rats; PE-11: preeclamptic rats at GD11; PE-19: preeclamptic rats at GD19.

In order to analyze whether NADPH oxidase was responsible for the overproduction of O_2_^●−^ that was observed in DHE-based experiments, the activity of this enzyme was assayed using inhibitors of different potential sources of O_2_^●−^, as well as inhibitors of various NOX isoforms. As shown in Figure 5A, NADPH oxidase activity was 63% higher in placental homogenates from PE-19 compared to the NP group. Interestingly, while the excess O_2_^●−^ was completely counteracted by DPI (suggesting a major involvement of NADPH oxidase in this sense), a significant (27%) decrease in superoxide levels was also found in PE animals after preincubation with ROT. Concerning specific NOX inhibitors, moderate reductions were obtained when placental homogenates were preincubated with ML171 and GKT136901 (17% and 34%, respectively), whereas preincubation with VAS2870 resulted in a massive reduction in O_2_^●−^ production, and led to values that were even lower than those measured in the NP group. In kidney samples (Figure 5B), the activity of NADPH oxidase augmented by 70% and 150%, respectively, in the PE-11 and PE-19 groups. Again, DPI restored O_2_^●−^ generation back to normal. However, contrarily to placental homogenates, ROT did not exhibit a significant inhibitory action towards O_2_^●−^ overproduction in preeclamptic animals. In turn, the use of NOX inhibitors ML171 (specific NOX1 inhibitor) and GKT136901 (NOX1/NOX4 inhibitor) returned reductions in O_2_^●−^ generation of 21% and 46%, respectively, while VAS2870 (pan-NADPH oxidase inhibitor) returned values in PE rats to those in NP rats.

Regarding the expressions of NOX isoforms, sFlt1-infused preeclamptic animals showed significant up-regulations of NOX1 (3.56-/1.73-fold at gene/protein level, respectively), NOX2 (3.89-/1.96-fold), and NOX4 (2.71-/1.70-fold) in the placenta (Figure 5C). Similar experiments in the kidney demonstrated relevant increases in the gene/protein expressions of NOX1 (2.33-/1.69-fold, respectively), NOX2 (1.97-/1.57-fold), and NOX4 (2.97-/2.46-fold) in the PE-19 group compared to NP rats (Figure 5D).

**Figure 6:**
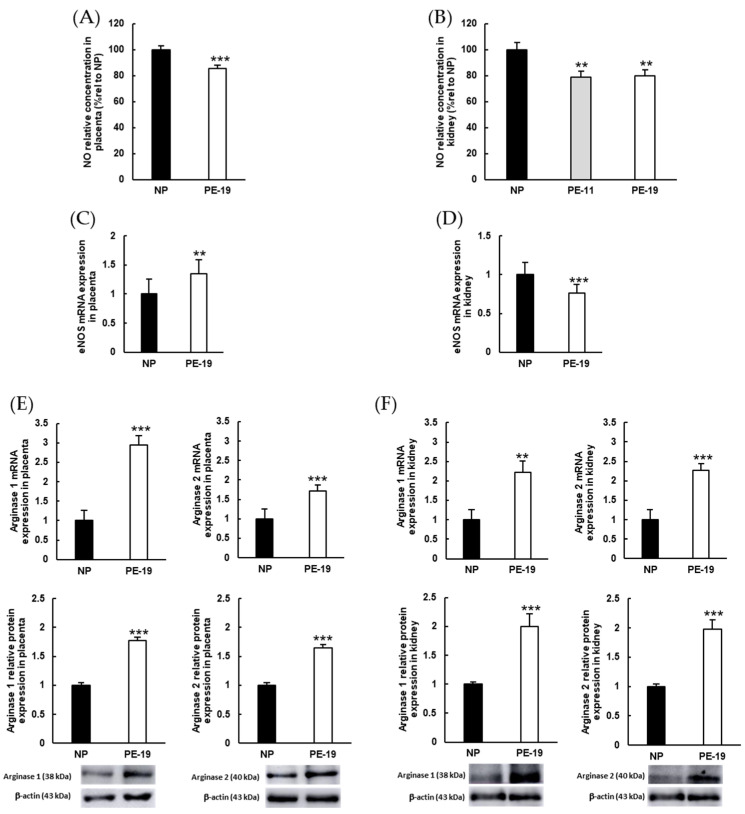
**Parameters related to NO metabolism in the placenta and kidney.** (**A**,**B**) NO relative concentrations estimated in placental and renal cortex homogenates. (**C**,**D**) eNOS gene expressions and (**E**,**F**) mRNA (upper charts) and protein (lower charts) expressions of arginase isoforms 1 and 2 in the placenta and kidney, respectively. The quantitative fold changes in gene expressions were determined relative to the corresponding values for the glyceraldehyde-3-phosphate dehydrogenase (GAPDH) housekeeping gene. Values are expressed as mean ± SEM of at least six animals per group: ** *p* < 0.01, *** *p* < 0.001 vs. NP. NP: normal pregnant rats; PE-11: preeclamptic rats at GD11; PE-19: preeclamptic rats at GD19.

Figure 6 gathers some parameters related to NO bioavailability, given the relevance of this regulatory molecule in the setting of oxidative stress and O_2_^●−^ generation by the NADPH oxidase system. A significant decrease in NO concentration was observed in preeclamptic placentas (15%) and kidneys (20%, both in PE-11 and PE-19) relative to NP rats (Figure 6A,B). On the other hand, the endothelial isoform of NOS (eNOS) was up-regulated (35% increase) in the placenta but repressed (24% decrease) in the kidney (Figure 6C,D). Arginase (i.e., the enzyme that induces degradation of the eNOS substrate, L-arginine) isoforms 1 and 2 were clearly overexpressed in both placental (Figure 6E) and renal cortex (Figure 6F) homogenates from sFlt1-treated rats (PE-19 group). Thus, the respective values for mRNA/protein expressions of arginase 1 increased 2.94/1.77 times in the placenta and 2.22/1.99 times in the kidney; and arginase 2 showed respective increments of 1.72-/1.65-fold (placenta) and 2.27-/1.97-fold (kidney).

### 3.6. Preeclampsia Modifies Enzymatic Antioxidant Defense Systems

**Figure 7:**
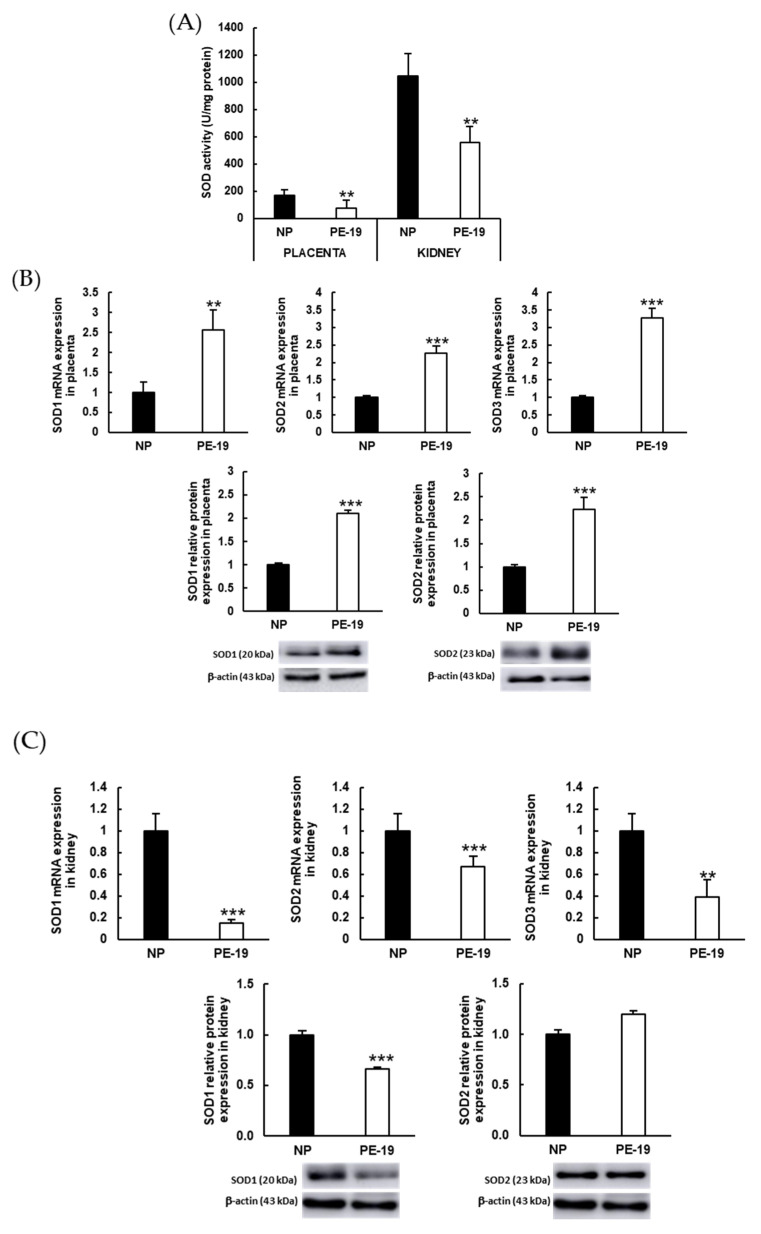

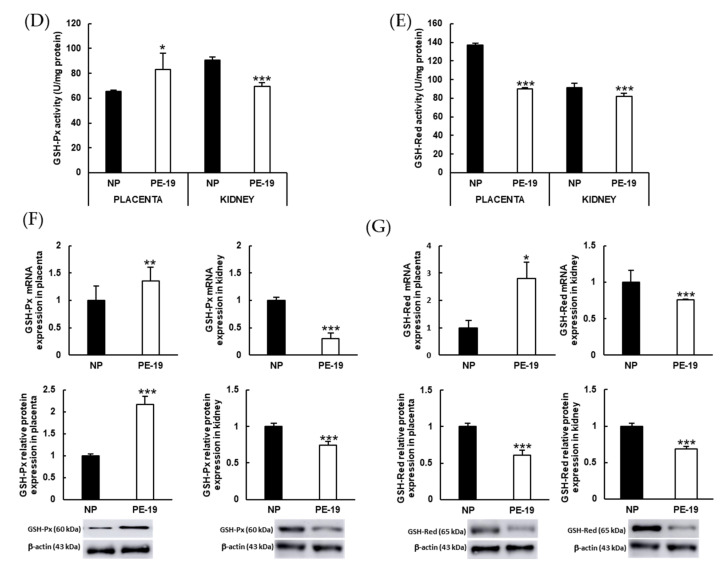
**Antioxidant enzyme activities and expressions in the placenta and kidney.** (**A**) Superoxide dismutase activities in placental and renal cortex homogenates. (**B**,**C**) Gene (upper charts) and protein (lower charts) expressions of SOD isoforms (SOD1, SOD2, and SOD3) in the placenta and kidney, respectively. (**D**) Glutathione peroxidase (GSH-Px) and (**E**) glutathione reductase (GSH-Red) activities in placental and renal cortex homogenates. (**F**,**G**) Gene (upper charts) and protein (lower charts) expressions of GSH-Px and GSH-Red, respectively, in the placenta (left charts) and kidney (right charts). The quantitative fold changes in gene expressions were determined relative to the corresponding values for the glyceraldehyde-3-phosphate dehydrogenase (GAPDH) housekeeping gene. Values are expressed as mean ± SEM of at least six animals per group: * *p* < 0.05, ** *p* < 0.01, *** *p* < 0.001 vs. NP. NP: normal pregnant rats; PE-19: preeclamptic rats at GD19; U: enzyme units.

A deep reduction in the total activity of SOD enzymes was found in both placentas and kidneys from preeclamptic animals when compared with the NP group (Figure 7A). Figure 7B depicts an evident overexpression of placental SOD isoforms in the PE-19 group compared to NP animals (2.57-/2.1-fold for SOD1, 2.27-/2.22-fold for SOD2, and 3.27-fold for SOD3 gene/protein expressions, respectively). On the contrary, SOD was markedly downregulated in the kidneys of preeclamptic animals, with the sole exception of SOD2 protein expression (Figure 7C).

Similar experiments were carried out for the antioxidant enzymes GSH-Px and GSH-Red. In this case, the preeclamptic phenotype displayed a significant (26%) increase in GSH-Px activity in the placenta together with a 30% reduction in the renal cortex (Figure 7D). A different pattern was observed for GSH-Red activity, which was lower in the PE-19 than in the NP group in both placental (34% reduction) and kidney (10% reduction) homogenates (Figure 7E). In turn, preeclamptic placentas showed up-regulation of GSH-Px gene/protein expressions (1.36-/2.16-fold, respectively), whereas GSH-Red expression was only reduced (40%) for protein expression (Figure 7F,G). In the kidney, both enzymes were down-regulated in preeclamptic animals when compared to normal pregnancies (70/26% for GSH-Px, and 25/32% for GSH-Red mRNA/protein expression, respectively).

### 3.7. Inflammation Biomarkers in the sFlt1-dependent Model of Preeclampsia

**Figure 8:**
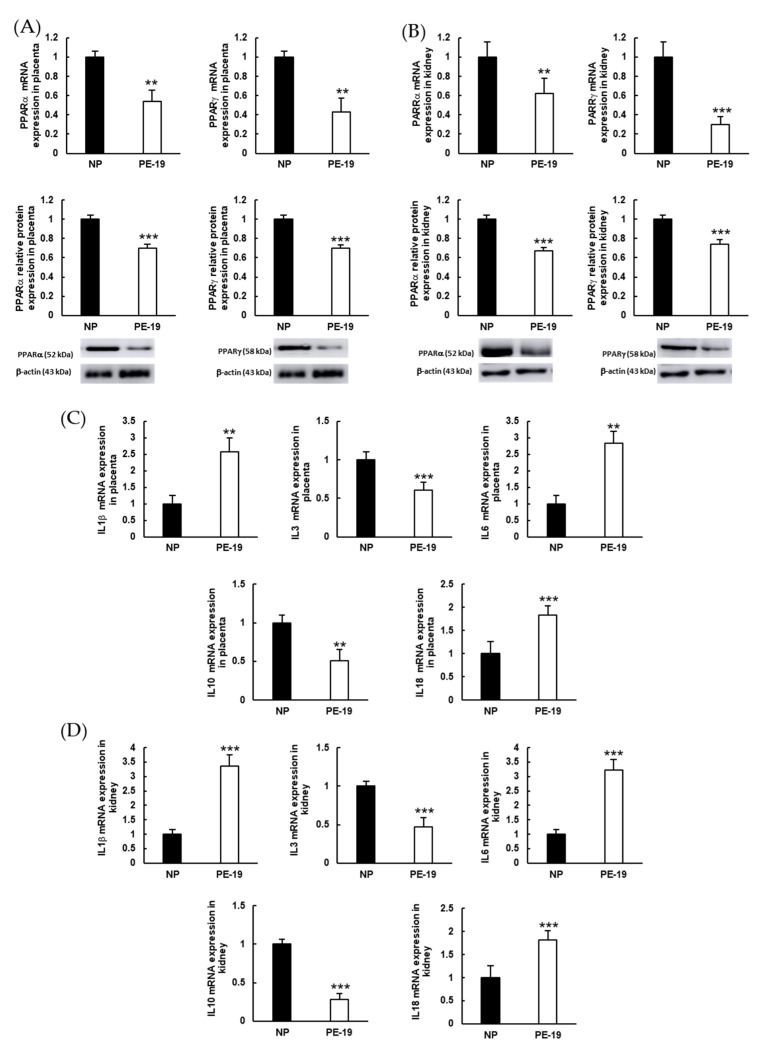
**Expressions of inflammation biomarkers in the placenta and kidney.** (**A**,**B**) Gene (upper charts) and protein (lower charts) expressions of peroxisome proliferator-activated receptors (PPAR) α (left charts) and PPARγ (right charts) in the placenta and kidney, respectively. (**C**,**D**) mRNA expressions of proinflammatory (IL1β, IL6, and IL18) and anti-inflammatory (IL3 and IL10) cytokines in the placenta and kidney, respectively. The quantitative fold changes in gene expressions were determined relative to the corresponding values for the glyceraldehyde-3-phosphate dehydrogenase (GAPDH) housekeeping gene. Values are expressed as mean ± SEM of at least six animals per group: ** *p* < 0.01, *** *p* < 0.001 vs. NP. NP: normal pregnant rats; PE-19: preeclamptic rats at GD19.

PE-19 animals presented significant depletion for PPAR gene/protein expression in the placenta (46/30% and 57/30% for PPARα and PPARγ, respectively; Figure 8A), and also in the kidney (38/32% and 70/26% for PPARα and PPARγ, respectively; Figure 8B), in relation to the NP group. Additionally, sFlt1 infusion resulted in substantial over expressions of the proinflammatory biomarkers interleukin (IL)1β (2.57-fold), IL6 (2.84-fold), and IL18 (1.84-fold) in the placenta, while down-regulating the anti-inflammatory IL3 (39% reduction) and IL10 (49% reduction; Figure 8C). Similar results were reproduced in preeclamptic kidneys (Figure 8D), where proinflammatory markers reached values 3.35, 3.23, and 1.82 times higher for IL1β, IL6, and IL18, respectively; and the expressions of anti-inflammatory IL3 and IL10 diminished by 53% and 72%, respectively.

## 4. Discussion

The data reported in this study support a role for NADPH oxidase in the development of oxidative stress and related inflammatory events in a rat model of early-onset preeclampsia (PE) induced by sFlt1. As previously reported, we found an increase in maternal blood pressure and a reduction in fetal and placental weight at preterm (GD19) in sFlt1-administered animals compared to the control group of rats with a normal pregnancy [26,27]. Besides hypertension, our animal model of PE presented with remarkable proteinuria, thus displaying at least two of the typical conditions that characterize this gestational disease. Similar findings have been regularly reported in others animal models of PE [28], as well as in preeclamptic patients with high sFlt1 concentration [29].

Current difficulties to establish criteria for the accurate and timely diagnosis of PE have led to continuous research in trying to find plausible molecular biomarkers that emerge early in this disorder. In this context, the ratio sFlt1/PlGF appears to be an excellent and effective indicator, not only at the clinical level [30] but also in different animal models of PE, as well as in other hypertensive gestational syndromes [26,31,32]. Excessive release of the antiangiogenic soluble endoglin is also considered another hallmark of PE [33]. The role of sFlt1 and soluble endoglin in the pathogenesis of PE is well accepted, especially in the development of the maternal syndrome observed in this disease [34,35]. Therefore, increases in the sFlt1/PlGF ratio and endoglin expression found in key target organs (placenta and kidney) in our PE animals at GD19 support the validity of a PE-like syndrome in our experimental conditions. Interestingly, we found an altered expression of sFlt1 and PlGF in the kidney of PE rats as early as at GD11 (i.e., only four days after exposure to high levels of sFlt1), which was already paralleled by proteinuria. This finding may suggest an early onset of antiangiogenic factor-mediated organ failure in this animal model of PE.

The placenta and the kidney are major organs affected by PE, with the former postulated as the key core in its development. Thus, morphological alterations have been reported in both the placenta and kidney under preeclamptic conditions [36,37], and histopathological examination of the kidney has been suggested as a predictive tool for prognostic purposes [38]. Collagen deposition in the placenta has been found in the reduced uteroplacental perfusion pressure (RUPP) preeclampsia animal model [39], and excess collagen deposition originated preeclampsia-like features in pregnant mice [40]. Interestingly, serum from PE patients has been shown to contain vasoactive substances that induce deposition of extracellular matrix components including collagen [41]. Despite these findings, the specific roles of the fibrotic placenta and collagen in the pathogenesis of preeclampsia remain unclear. In the current study, in addition to the placenta, we also found excess collagen deposition in the kidney of PE animals compared to normal pregnant rats; not only was this alteration seen at preterm (GD19), but also at GD11, which might be related to renal overexpression of sFlt1 at this stage.

Preeclampsia conditions have been associated with the presence of oxidative imbalance that exacerbates endothelial cell dysfunction, leading to adverse pregnancy outcomes [13]. In this sense, the local overproduction of O_2_^●−^ reported in the current study in the placenta and kidney of early-onset preeclamptic rats appears to be related to an induction of NADPH oxidase (NOX) activity and expression. Similar results were previously reported in sFlt1-induced hypertension in pregnant rats [42]. Other authors, however, found no alterations in the expressions of placental NOXes in preeclamptic patients when compared with normal pregnancies [43]. In the kidney, both superoxide-dependent DHE staining and NADPH oxidase activity, which have been proposed as the main sources of O_2_^●−^ in first-trimester pregnancies [17], were found to be increased in PE rats from GD11 (i.e., shortly after the beginning of sFlt1 infusion). Therefore, this NADPH system dysregulation may be associated with the antiangiogenic factor imbalance already present at GD11.

Regarding NADPH oxidase isoforms, the use of specific NOX inhibitors, along with NOX isoform expression analyses, indicated that O_2_^●−^ overproduction in the placenta of preeclamptic animals was mainly dependent on the NOX2 isoform, while NOX4 seems to be more relevant in the kidney. These differential expressions of NOXes may provide useful biomarkers and targets for PE treatment through the selective blockade of these isoforms by specific drugs. Previous results have shown NOX1 upregulation in syncytiotrophoblast [44] and increased placental NOX2 expression under different preeclamptic environments [45,46]; such findings are also extendable to other components of the NADPH oxidase system [47].

Nitric oxide plays an important role in pregnancy because it participates in the development and vascular remodeling of the placenta. An increase in ROS leads to high production of peroxynitrite, with a reduction in NO levels and impaired vascular function and placental perfusion [48]. In the current study, we found reduced NO levels in both placental and renal homogenates from PE animals. In the kidney, the concentration of NO was similarly altered in PE-11 and PE-19 groups, suggesting that a quick reduction in NO bioavailability occurs from the beginning of sFlt1 exposure and remains throughout pregnancy in PE animals. Although these results might be due to changes in eNOS expression, as previously reported [49,50], our findings in this regard were inconclusive because eNOS appeared to be up-regulated in the placenta while it was down-regulated in the kidney of sFlt1-treated rats. Alternatively, NO depletion could be secondary to the increase found in PE rats in the expression of arginase (a key enzyme that regulates NO synthases through depletion of its substrate), which is especially relevant in the hypertensive context [51]. Decreased NO levels have been found in the kidney and serum in response to sFlt1 in pregnant rats [52] and in the L-NAME-induced (i.e., NO-depleted) PE-like rat model [53]. In addition, excessive NADPH oxidase activity and ROS production, along with arginase overexpression (as observed in PE rats), leads to eNOS uncoupling and switches its activity from NO to O_2_^●−^ production. This eNOS uncoupling may eventually potentiate oxidative imbalance, thus reproducing in rodents the alterations in NO metabolism displayed in human preeclampsia [54]. Consistent with previous studies in humans and in animal models [55,56,57,58], the overexpression of nitrotyrosine (a well-recognized oxidative biomarker) in the placenta and kidney of preeclamptic animals reinforces alterations in NO signaling in the context of this syndrome [59].

Experiments on the activity and expression of antioxidant enzymes confirmed the presence of oxidative imbalance in our model of early-onset PE. Thus, the activity of SOD was significantly depleted in both the placenta and kidney of preeclamptic animals. Interestingly, SOD isoforms, namely SOD1 (cytosolic), SOD2 (mitochondrial), and SOD3 (extracellular), were upregulated in the placenta following sFlt1 exposure, while the opposite pattern was found in the kidney. Reductions in serum and placental SOD activity and placental SOD2 expression have recently been reported in a mouse model of PE [60]. Contrarily, Roland et al. [61] found an up-regulation of SOD isoforms in the placentas of PE patients, suggesting a possible implication in the defense against oxidative stress in this context. An increase in SOD isoforms has also been described in HUVEC (human umbilical vein endothelial cells) incubated with plasma from preeclamptic women [62].

Recently, several studies have evidenced the role of the mitochondria as an important intracellular source of ROS in the pathophysiology of PE [43]. For instance, a mitochondria-targeted antioxidant therapy showed promise in improving fetal outcomes in a mouse model of PE [63]. In our study, we found a significant decrease in superoxide anion generation in the preeclamptic placenta following incubation with rotenone (an inhibitor of the mitochondrial electron transport chain), which supports the notion that mitochondria might contribute substantially to superoxide anion overproduction in placental tissue in the setting of PE. This finding, together with the increase found in the placental SOD2 isoform, suggests the presence of mitochondrial dysfunction in our rodent model of PE, as previously reported [64]. Since these changes were not observed in the kidney, mitochondrial dysfunction seems to be mainly relevant in the placenta, at least in our experimental conditions.

The enzyme GSH-Px appeared to be differentially regulated in the placenta and kidney of PE animals. In turn, GSH-Red was rather defective in both tissues despite higher mRNA expression in the placenta, interestingly. Huang et al. [60] and Kaur et al. [65] related a decrease found in GSH-Px that was obtained from the serum of PE mice and from the plasma of PE women. On the other hand, both GSH-Px and GSH-Red were stimulated in endothelial cells incubated with plasma from PE patients [66]. Taken together, these results indicate that modifications in the activity and expressions of antioxidant enzymes in PE may be tissue dependent and are also variable depending on the specific experimental design used to study the disease.

Along with oxidative stress, inflammation has been widely studied as a key event in the pathophysiological mechanisms surrounding preeclampsia [67]. In addition to regulating glucose and lipid metabolism [68], PPARs can also help reduce excessive inflammation, as they regulate a wide variety of genes involved not only in inflammation but also in differentiation, proliferation, and metabolic pathways [68]. As a consequence, PPARs can counteract the proinflammatory actions of IL-6 and TNFα, as elucidated in experiments carried out with PPAR agonists [69,70]. Our model of PE based on excess sFlt1 displayed reduced protein and mRNA expressions of PPARα and PPARγ in kidney and placental tissues. Previous reports showed that preeclampsia can induce pathologic inflammation in the placenta of pregnant rats by reducing PPAR expression [71]. Moreover, recent findings suggest a critical role for PPARs in mitochondrial regulation [64], and it is known that PPARs can regulate excessive ROS production by suppressing NADPH oxidase [64]. Therefore, the reductions in PPAR expression are generally associated with oxidative and inflammatory events. In this sense, our PE model clearly developed an inflammatory profile in placental and renal tissues, as demonstrated by the presence of increased proinflammatory biomarkers (IL1β, IL6, and IL18) and low levels of anti-inflammatory IL3 and IL10. Similar results have been reported in the serum and placenta of rats with L-NAME-induced PE [53,72,73], and also in preeclamptic mice [74]. It should be noted here that there is some controversy about the relevance of IL6 in the context of preeclampsia [75,76]. On the other hand, an increase in IL18 has been well correlated with oxidative imbalance in preeclamptic patients [77,78]. Concerning anti-inflammatory cytokines, other authors have also reported a decrease in placental IL10 in PE women [79]. IL10 deficiency may contribute to the pathogenesis of PE, since this cytokine appears to be relevant in regulating arterial blood pressure in early primate pregnancy [80]; and IL10 is also necessary to maintain blood pressure and avoid inflammation and endothelial dysfunction in hypertensive pregnant rats [81]. Overall, these results support the utility of the sFlt1-induced PE-like phenotype and warrant further animal studies that may help understand the precise mechanisms involved in the development of inflammatory processes in preeclampsia.

## 5. Conclusions

In order to understand the multiple and complex mechanisms involved in the pathophysiology of preeclampsia, we need to broaden current research fields using animal models that try to mimic as far as possible the characteristics of the syndrome that develops in humans. The present study sheds light on the deleterious role of the enzyme NADPH oxidase in the setting of this disease by using a preeclampsia-like model based on premature infusion of the antiangiogenic factor sFlt1. Excessive O_2_^●−^ production and alterations in NO metabolism developed shortly after initiating sFlt1 administration. NOX2 and NOX4, respectively, appear to be particularly involved in the oxidative imbalance that occurs in the placenta and kidney. Furthermore, mitochondrial dysfunction may also participate in placental ROS overproduction during PE pregnancies. Along with oxidative stress, free radicals contribute to establishing an inflammatory imbalance in this model of preeclampsia. Our findings support the utility of animal models based on sFlt1 overload and reinforce the importance of the NADPH oxidase system in the pathogenesis of preeclampsia by mediating oxidative and (possibly) inflammatory processes that contribute to disease progression in key target organs, such as the placenta and the kidney.

## Figures and Tables

**Table 1:** Genes analyzed by RT-PCR.

Gene	Forward Primer (5’→3’)	Reverse Primer (5’→3’)	Accession Number
sFlt1	CAAGGGACTCTACACTTGTC	CCGAATAGCGAGCAGATTTC	AF157595.1
PlGF	CAGCCAACATCACTATGCAG	TCCTCTGAGTGGCTGGTTA	NM_053595.2
Endoglin	CCAAGGCTGCCACTTGG	GATGCTGTGGTTGGTAC	NM_001010968.3
Nox1	GGTTGGGGCTGAACATTTTTC	TCGACACACAGGAATCAGGAT	NM_172203.2
Nox2	CCCTTTGGTACAGCCAGTGAAGAT	CAATCCCACGTCCCACTAACATCA	AF298656.3
Nox4	GGATCACAGAAGGTCCCTAGC	AGAAGTTCAGGGCGTTCACC	NM_053524.1
Arginase 1	ATTTCGGTGGTTTAAGGTAGTCAG	TACAAGACAGGGCTACTTTCAG	NM_017134.3
Arginase 2	CCTCTTCCTCTTGCCAATCAG	CAGCCTCTTTCCTTTCTCATCAG	U90887.1
SOD1	AATGTGTCCATTGAAGATCGTGTGA	GCTTCCAGCATTTCCAGTCTTTGTA	NM_017050.1
SOD2	AGGGCCTGTCCCATGATGTC	AGAAACCCGTTTGCCTCTACTGAA	NM_017051.2
SOD3	GGGTCTGTCCTGTACTTCACCAGAG	CTGACATGGTCCAGGTGACAGAG	NM_012880.2
GSH-Px	GGAGAATGGCAAGAATGAAGA	CCGCAGGAAGGTAAAGAG	NM_030826.4
GSH-Red	GGAAACTCGCCCATAGACTT	CCAACCACCTTCTCCTCTTT	NM_053906.2
PPARα	ACGATGCTGTCCTCCTTGATG	GCGTCTGACTCGGTCTTCTTG	NM_013196.2
PPARγ	CTGGCCATATTTATAGCTGTCATTATT	AGCAGGTTGTCTTGGATGTCCT	Y12882.2
IL1β	GAGGCTGACAGACCCCAAAAGAT	GCACGAGGCATTTTTGTTGTTCA	NM_031512.2
IL3	CTTGATGTCCATTGTGTCCTGAG	TCCTGATGCTCTTCCACCAG	NM_031513.2
IL6	TTCTGCAAGTGCATCATCGT	CTCTGCAAGAGACTTCCATCC	NM_012589.2
IL10	AGGCCATTCCATCCGGGGTGA	AGGCAGCCCTCAGCTCTCGG	NM_012854.2
IL18	TCCTTCACAGAGAGGGTCACA	GACAGCCTGTGTTCGAGGAT	NM_019165.2
GAPDH	GCCAAAAGGGTCATCATCTCCGC	GGATGACCTTGCCCACAGCCTTG	NM_017008.4

**Table 2:** Antibodies used for Western blotting.

1st Antibody	Origin	Dilution	2nd Antibody	Dilution	Reference
Anti-VEGFR1	Rabbit monoclonal	1:2000	Goat Anti-Rabbit	1:4000	Epitomics-Abcam, Burlingame, CA
Anti-NOX1	Mouse monoclonal	1:1000	Goat Anti-Mouse	1:2000	SCB ^1^
Anti-NOX2	Rabbit monoclonal	1:9000	Goat Anti-Rabbit	1:10,000	Epitomics-Abcam
Anti-NOX4	Rabbit monoclonal	1:9000	Goat Anti-Rabbit	1:10,000	Epitomics-Abcam
Anti-nitrotyrosine	Mouse Monoclonal	1:1000	Goat Anti-Mouse	1:2000	SCB
Anti-arginase 1	Mouse monoclonal	1:1000	Goat Anti-Mouse	1:2000	SCB
Anti-arginase 2	Mouse monoclonal	1:1000	Goat Anti-Mouse	1:2000	SCB
Anti-GSH-Px	Mouse monoclonal	1:1000	Goat Anti-Mouse	1:4000	SCB
Anti-GSH-Red	Rabbit polyclonal	1:5000	Goat Anti-Rabbit	1:8000	SCB
Anti-SOD-1	Mouse monoclonal	1:1000	Goat Anti-Mouse	1:2000	SCB
Anti-SOD-2	Mouse monoclonal	1:1000	Goat Anti-Mouse	1:2000	SCB
Anti-PPARα	Mouse monoclonal	1:2000	Goat Anti-Mouse	1:4000	SCB
Anti-PPARγ	Mouse monoclonal	1:2000	Goat Anti-Mouse	1:4000	SCB
Anti-β-Actin	Mouse monoclonal	1:20,000	Goat Anti-Mouse	1:30,000	SCB

^1^ SCB = Santa Cruz Biotechnology (Santa Cruz, CA, USA).

**Table 3:** General parameters measured in the different experimental groups at the end of the treatment.

Parameters	NP	PE-19
Animal weight (g)	278 ± 3.9	267 ± 4.3
Embryo weight (g)	2.29 ± 0.12	1.54 ± 0.10 ***
Average no. embryos	10	10
Kidney weight (g)	0.71 ± 0.02	0.75 ± 0.02
Placenta weight (g)	0.68 ± 0.021	0.44 ± 0.066 **

Values are expressed as mean ± SEM of at least six animals per group: ** *p* < 0.01, *** *p* < 0.001 vs. NP. NP: normal pregnant; PE-19: preeclamptic rats at GD19.

## Data Availability

The data presented in this study are available in article.
